# What We Owe to Experiments on Animals

**Published:** 1903-12-26

**Authors:** Stephen Paget


### Hospital Clinics and Medical Progress.

### WHAT WE OWE TO EXPERIMENTS ON ANIMALS.

By Stephen Paget.

(*Continued from page* 204.)

### *II.—PATHOLOGY, BACTERIOLOGY, AND THERAPEUTICS.*

#### I.—Inflammation, Suppuration, etc.

Before bacteriology, the processes of inflammation and suppuration had been studied so carefully that nothing more could be done till Pasteur came. And, of all those earlier studies, the most valuable had been the direct observation, under the microscope, of every stage of inflammation in the web of the foot of the frog.

The work of Pasteur interpreted a whole host of facts that were waiting for him. It not only interpreted them, it also compelled men to believe its teaching, and to act on that belief. Take, for example, the contrast between Semmelweis and Pasteur:[1] between the man who could not compel belief, and the man who did. Semmelweis, in 1847, by a long series of clinical observations, and, so far as we know, without any experiments on animals, discovered that the ghastly mortality from puerperal fever in the great general hospital of Vienna, was due to direct infection, such as might occur in the examination of patients. "Not only," he said, "can the particles from dead bodies generate puerperal fever, but any decomposed material from the living body can also generate it, and so can air contaminated by such materials." Forthwith, he isolated all infected cases, he made the use of disinfectants compulsory and universal; and the deaths, which in May, 1847, had stood at the terrible figure of 12·24 per cent., fell in December, 1847, to 3 04, and in 1848 to 1·27. Then came furious opposition: he was bullied by the university authorities, deprived of his professorship, driven out of Vienna, driven out of his senses; he went mad, he only came back to Vienna to die in an asylum there, having fought for 18 years in vain. It was indeed in vain; his work had to be done all over again. The year before his death, out of 1,350 women in the Maternity Hospital of Paris, 310 died; in two months, just after his death, out of 103 women, 28 died.

Why did he fail? How was it that his enemies were able to ruin him? It was because he could not show to them, there in a test-tube, there in the tissues of an inoculated rabbit, there in the bodies of patients, the thing itself, the microscopic organisms that are the cause of the disease. He could show them everything else, and he did: how their patients died, and why they died, and where the fault lay. For want of bacteriology, for want of inoculation-experiments, he was defeated, talked down, hustled out of life.

Now, take a story of Pasteur. In 1878, when Semmelweis had been for 13 years in his grave, there was a discussion at the French Academy of Medicine on this same subject of puerperal fever. Somebody was making a long discourse on the causes of these mysterious visitations. Pasteur could not keep silence, and exclaimed off-hand—"The cause of the epidemic has nothing to do with all that; it is the doctor and his assistants who convey the microbe from a woman who is diseased to a woman who is healthy." The orator answered, "I am very much afraid that nobody will ever find that microbe." Pasteur jumped up, went to the blackboard, drew the *streptococcus* on it, and said, "There! That's what it looks like!" *See how far we have come*, says one of Pasteur's greatest pupils, *from the old metaphysical ideas about virulence, to these microbes that we can turn this way or that way—stuff so plastic that a man can work on it, and fashion it as he likes.* That is the revolutionary force of bacteriology. The thing itself, the one and only exciting cause of this or that disease, is here, in a test-tube, in a rabbit, in a drop of blood under the microscope: *Tenez, Voici sa figure—That's what it looks like.* And the men of science know the habits of this or that microbe, its phases and its ways, its likes and its dislikes, and all the influences on it of temperature, air, light, and chemistry; they can increase or diminish or destroy its virulence, foretell its actions and its products, and the whole course of its invasion, multiplication, and dispersal through the body, and what kind and degree of resistance it will meet there. It is part of the day's work to determine these infinitesimal changes in these invisible bacteria; and every year the wonder and the accuracy of the work are increased.

From Semmelweis to Pasteur—from Pasteur to Lister. In the book "Experiments on Animals," Lord Lister himself was pleased to write, with much else, this sentence: "The antiseptic method was based on bacteriology, resting as it did on the proof afforded by Pasteur that putrefaction was caused by bacteria and not by the oxygen of the air, as had been previously believed." Again, in his Huxley Lecture, October, 1900, having spoken of his early study of pyæmia, he goes on:—

"While these investigations into the nature of pyæmia were proceeding I was doing my utmost against that deadly scourge. . . . I did my best, by local measures, to diminish the risk of communicating contagion from one wound to another. I freely employed antiseptic washes, and I had on the tables of my wards piles of clean towels to be used for drying my hands and those of my assistants after washing them, as I insisted should invariably be done, in passing from one dressing to another. But all my efforts proved abortive, as I could hardly wonder, when I believed, with chemists generally, that putrefaction was caused by the oxygen of the air.

"It will thus be seen that I was prepared to welcome Pasteur's demonstration that putrefaction like other true fermentations, is caused by microbes growing in the putrescible substance. Thus was

[1] For Semmelweis see "Experiments on Animals" (John Murray, 1903) p. 90. For the story about Pasteur, see "Roux, Œuvre Médicale de Pasteur, Agenda du Chimiste," 1896, p. 528; also Mrs. Devonshire's translation of M. Vallery-Radot's beautifully written "Life of Pasteur," vol. ii, p. 89.

presented a new problem—not to exclude oxygen from the wounds, which was impossible, but to protect them from the living causes of decomposition. . . . To apply that principle, so as to ensure the greatest safety with the least attendant disadvantages, has been my chief life work."

There is no need here to consider the difference between aseptic surgery (sterilization) and antiseptic surgery. From the point of view of science, both of them are based on bacteriology, and bacteriology is based on experiments on animals. From the point of view of practice, aseptic surgery and antiseptic surgery are as inseparable, in the phrase of Aristotle, as the convexity and concavity of a curve.

It is often said by the opponents of all experiments on animals that excellent results were obtained by one well known surgeon, though he did not follow the rules of "Listerism." But the performance, under favourable conditions, of certain major operations, with an incision through clean and healthy tissues, is only one part of surgery. What about the thousands of surgical cases that come to hospital with dirty lacerated wounds, crushed hands, sloughing tissues, foul sores, and all such infected and infecting surfaces; the "casualties" and the outpatients? What about the victory that has been won over "hospital diseases"—pyæmia, septicæmia, cellulitis, erysipelas, and tetanus? Or take the present treatment of empyema, or of compound fracture, or of a suppurating joint. It is nothing that the patients of A or B or C did well without those "antiseptic precautions" that are now the rule in our laboratories to prevent inflammation even in rabbits after operation. The thing is, that these precautions are observed all the world over, by all sorts and conditions of men; and that the saving of limbs and of lives, year in year out, is past all measure. But let us put it, for the whole world, at 50,000 lives every year, and 50,000 limbs. That is well below the mark.

### II.—Anthrax.

Anthrax, or *charbon*, is an acute infective fever due to a micro-organism, the *bacillus anthracis*. A quarter of a century ago it was terribly common among sheep and cattle. In man, it occurs among wool-sorters, hide-dressers, rag-pickers, and butchers. The sore at the point of inoculation is called malignant pustule, and the infection by inhalation into the lungs is called wool-sorters' disease. Happily, anthrax is very rare among men.

It was the first disease ever studied by the methods of bacteriology; no words can give any idea of the infinite importance, to all mankind, of the results that were achieved. The micro-organisms had been seen, sixty years ago, in a drop of blood from a dead sheep; but they had been taken for some odd sort of blood-crystals. Koch, thirty years later, had isolated them, had made cultures of them, and had reproduced the disease in animals by inoculation with the cultures. Then came Pasteur's work, and his discovery in 1881 of a preventive vaccine for flocks and herds. The story of this tremendous crisis in Pasteur's life must be read in M. Vallery-Radot's book; and no man need ever wish for better reading. If the final public test-experiments at Pouilly-le-Fort on the two groups of sheep were successful, it would mean not only the protection of millions of animals in years to come, but also the absolute and visible justification before the world of this new sort of science; as Roux said of this first triumph of bacteriology:—

"The immediate result of Pasteur's vaccinations is their least merit. They have given men absolute faith in a science that could show such good works, they have started a movement that is irresistible; above all, they have set going the whole study of immunity, which is bringing us at last to a right way of treating infective diseases."

In the next eleven years, 1882–1893, in France alone, more than three million sheep were inoculated, and half a million cattle. In Italy, in one year, May 1897–April 1898, from one Institute in Milan, 165,000 tubes of vaccine were sent out. It is said that the method failed in Hungary 18 years ago, and was abolished by the Government of that remote country at that remote date; but, whatever may have happened in Hungary 18 years ago, it is in strange contrast with the results that happened in France at a much later period. They are given in a monograph by M. Chamberland, *Résultats pratiques des Vaccinations contre le Charbon et Rouget en France* (Annales de l'Institut Pasteur, March 1894). And they come to this—that the deaths from *charbon* among cattle fell from 5 per cent. to $\frac{1}{3}$ per cent., and among sheep from 10 per cent. to 1 per cent.; and the deaths from *rouget* among swine fell from 20 per cent. to $1\frac{1}{2}$ per cent.

Here is a good record, and it is not the only instance of the benefit that animals enjoy from experiments on animals. For every single animal that was killed in Pasteur's laboratories by inoculation with *charbon*, we must reckon that thousands of animals have been saved or safeguarded from that disease.

*(To be continued.)*

